# Insights to SARS-CoV-2 life cycle, pathophysiology, and rationalized treatments that target COVID-19 clinical complications

**DOI:** 10.1186/s12929-020-00703-5

**Published:** 2021-01-12

**Authors:** Ioannis P. Trougakos, Kimon Stamatelopoulos, Evangelos Terpos, Ourania E. Tsitsilonis, Evmorfia Aivalioti, Dimitrios Paraskevis, Efstathios Kastritis, George N. Pavlakis, Meletios A. Dimopoulos

**Affiliations:** 1 Department of Cell Biology and Biophysics, Faculty of Biology, National and Kapodistrian University of Athens, 15784 Athens, Greece; 2 Department of Clinical Therapeutics, School of Medicine, National and Kapodistrian University of Athens, 11528 Athens, Greece; 3 Department of Animal and Human Physiology, Faculty of Biology, National and Kapodistrian University of Athens, 15784 Athens, Greece; 4 Department of Hygiene, Epidemiology and Medical Statistics, School of Medicine, National and Kapodistrian University of Athens, 11527 Athens, Greece; 5 Human Retrovirus Section, National Cancer Institute, Frederick, MD 21702 USA

**Keywords:** ACE2, ARDS, COVID-19, SARS-CoV-2, TMPRSS2

## Abstract

**Background:**

Gaining further insights into SARS-CoV-2 routes of infection and the underlying pathobiology of COVID-19 will support the design of rational treatments targeting the life cycle of the virus and/or the adverse effects (e.g., multi-organ collapse) that are triggered by COVID-19-mediated adult respiratory distress syndrome (ARDS) and/or other pathologies.

**Main body:**

COVID-19 is a two-phase disease being marked by (*phase 1*) increased virus transmission and infection rates due to the wide expression of the main infection-related *ACE2*, *TMPRSS2* and *CTSB/L* human genes in tissues of the respiratory and gastrointestinal tract, as well as by (*phase 2*) host- and probably sex- and/or age-specific uncontrolled inflammatory immune responses which drive hyper-cytokinemia, aggressive inflammation and (due to broad organotropism of SARS-CoV-2) collateral tissue damage and systemic failure likely because of imbalanced ACE/ANGII/AT1R and ACE2/ANG(1–7)/MASR axes signaling.

**Conclusion:**

Here we discuss SARS-CoV-2 life cycle and a number of approaches aiming to suppress viral infection rates or propagation; increase virus antigen presentation in order to activate a robust and durable adaptive immune response from the host, and/or mitigate the ARDS-related “cytokine storm” and collateral tissue damage that triggers the severe life-threatening complications of COVID-19.

## Introduction

**Fig. 1:**
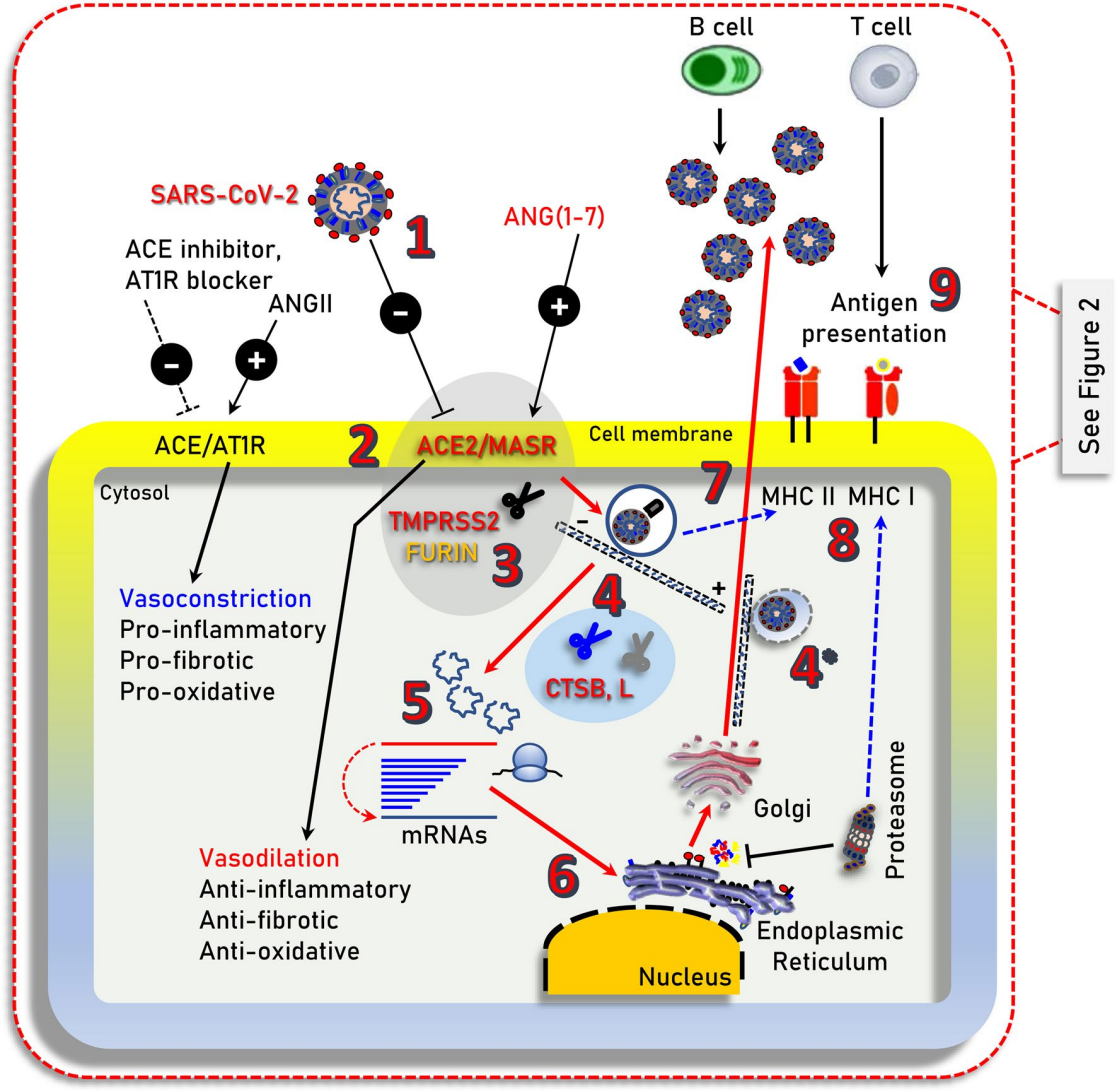
Illustration of the main cell signaling axes [i.e., ACE/ANGII/AT1R and ACE2/ANG(1–7)/MASR] and of other cellular components being involved in SARS-CoV-2 infection (i.e., TMPRSS2 or furin), endocytosis and replication. **1.** SARS-CoV-2 (extracellular); **2.** binding to ACE2; **3.** TMPRSS2 (or FURIN) priming; **4.** clathrin-mediated endocytosis (entry to early and acidic late -microtubule bound- endosomes) - 4* denotes endosomal compartments during exocytosis; **5, 6.** uncoating, genomic RNA release and viral-protein synthesis in free and endoplasmic reticulum-attached ribosomes; **7.** vesicle-mediated exocytosis; **8.** antigen presentation by endocytic compartments (MHC II) and proteasomes (MHC I); **9.** immune cell attraction and development of immunity or elimination of infected cells. MHC II/MHC I, Major Histocompatibility Complex class II, I. ┤inhibition, → induction

Coronavirus disease 2019 (COVID-19) is caused by severe acute respiratory syndrome coronavirus 2 (SARS-CoV-2) and has resulted in more than 1.45 million of deaths worldwide as of November 30, 2020. While the majority of SARS-CoV-2 infected patients will not require hospitalization, a minority will present with more severe symptoms requiring hospitalization and may experience severe life-threatening complications, including acute respiratory distress syndrome (ARDS), which may trigger a systemic multi-organ collapse [1]. Since SARS-CoV-2 is a new virus and there are few (e.g., Remdesivir; an antiviral drug initially used against hepatitis C virus) [2] anti-viral drugs that have been re-purposed for COVID-19 treatment [3], a better understanding of the underlying COVID-19 pathobiology is required in order to design prophylactic and/or therapeutic strategies. SARS-CoV-2 infects human cells by binding to the cell surface protein angiotensin-converting enzyme 2 (ACE2) through the Receptor Binding Domain (RBD) of its spike (S) protein (Fig. 1) [4]. In addition, the cellular transmembrane serine protease 2 (TMPRSS2) is required for the priming of the virus S protein [4, 5], while virus entry in the cell may also depend on the endosomal/lysosomal cysteine proteases cathepsin B and L (CTSB, CTSL) although their activity is likely not essential [4]. More recently, it was found that furin protease is also involved in the infection process since SARS-CoV-2 contains an unusual for coronaviruses furin cleavage site in the S protein [6], and that the cellular receptor neuropilin-1 (NRP1, binds furin-cleaved substrates) potentiates SARS-CoV-2 infectivity providing also a pathway into the central nervous system [7]; SARS-CoV-2 may also utilize the putative alternative receptor CD147 (expressed in high levels in the brain) to infect cerebral nervous system [8, 9].

## The critically balanced ACE/ANGII/AT1R and ACE2/ANG(1–7)/MASR axes

ACE2 is a main component of the renin-angiotensin system (RAS) which maintains fluid and salt balance, as well as blood pressure homeostasis [10]. Renin, angiotensinogen (AGT), angiotensin-converting enzyme (ACE), angiotensin II (ANGII) and the ANGII type 1 and type 2 receptors (AT2R1 and ATR2) (Fig. 1; AT2R is not shown) are major components of RAS. ACE generates ANGII which is a key effector peptide causing vasoconstriction. Overactivation of RAS has been implicated in the pathophysiology of atherosclerosis, heart failure, hypertension, diabetes, renovascular disorders, pulmonary hypertension, pneumonia, fibrosis, and sepsis [11, 12]. On the other hand, ACE2 which has considerable homology (40% identity and 61% similarity) to ACE metalloprotease [13, 14], functions as a negative regulator of the RAS system. Specifically, ACE2 reduces ANGII levels by cleaving it to the sorter ANG(1–7) peptide, which can then activate the vasodilation-promoting and anti-inflammatory MAS receptor (MASR) (Fig. 1) [15]. Furthermore, it has been reported that ACE2 links amino acid malnutrition to intestinal inflammation, as it is a key regulator of innate immunity, dietary amino acid homeostasis and gut microbial ecology [16]. Overall, the balance between the ACE/ANGII/AT1R and the opposing ACE2/ANG(1–7)/MASR axes is central in (among others) the physiological regulation of cardiovascular, blood pressure, neural and kidney functions [10, 12, 15].

It can be assumed that increased ACE2 expression or the co-expression at high levels of the ACE2, TMPRSS2 and CTSB/L proteins in SARS-CoV-2 targeted cells/tissues will correlate with higher risk of viral infection. Reportedly, the *ACE2*, *TMPRSS2* and *CTSB/L* genes/proteins are widely expressed in human tissues; being particularly enriched in kidney, heart, as well as in tissues of the respiratory and gastrointestinal tract [17]. The *ACE2* and *TMPRSS2* genes are minimally expressed in blood cells and tend to be co-regulated [17]; it was also found that the SARS-CoV-2 entry factors are expressed at high levels in nasal epithelial cells [18]. These observations suggest that even in the absence of underlying co-morbidities most vital human organs are potentially vulnerable to SARS-CoV-2 infection. It was also found that the *ACE2/TMPRSS2* genes are (among others) downregulated by tumor necrosis factor (TNF) and are induced by several pro-inflammatory conditions including Barrett's esophagus, gastric infection by *Helicobacter pylori*, obesity, diabetes, autoimmune diseases, as well as by viral infections, cigarette smoking, growth factors, interferons (IFNs) and androgens [17]. In support, ACE2 expression was stimulated by a *type I Interferon* (*IFN-a*) gene in human airway epithelial cells [19] and thus, SARS-CoV-2 could (indirectly) exploit IFN-driven upregulation of ACE2 to enhance infection rate in target tissues.

In pathologies like diabetes, obesity, hypertension, respiratory or cardiovascular disease which have all been found to associate with high-risk severe COVID-19 [20], the pro-inflammatory ACE/ANGII/AT1R axis is overactivated triggering the overexpression of the counteracting ACE2 pathway increasing thus SARS-CoV-2 available binding sites. In most cases these patients are treated with anti-hypertensive drugs including ACE inhibitors or ANGII receptor blockers (Fig. 1) [21]; consistently, prescription of anti-hypertensives was more frequent among patients with COVID-19 [22].

**Fig. 2:**
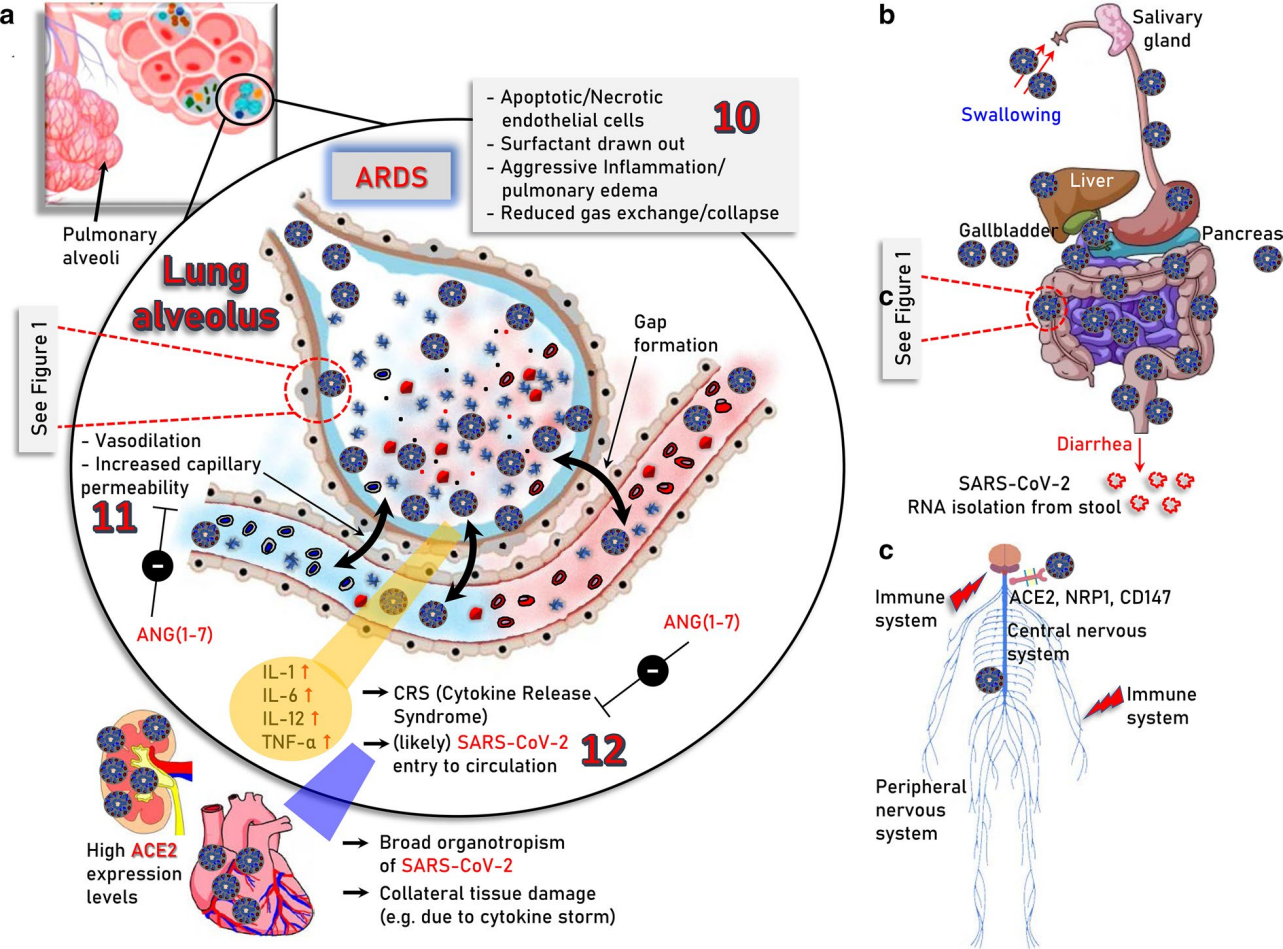
Major severe COVID-19 pathologies and infection routes. **a** The modules involved in, **10.** acute phase of SARS-CoV-2 infection in the lung (ARDS); **11.** vasodilation, increased capillary permeability, apoptosis/necrosis of endothelial cells as well as **12.** ARDS-induced “cytokine storm” and likely virus entry to the circulation which may then cause systemic failure due to broad organotropism in tissues expressing high levels of ACE2 (e.g., heart and kidneys) or the “cytokine storm”-related excessive inflammation, are indicated. **b** Sites of potentially SARS-CoV-2 infected organs in the alimentary tract of the digestive system and in accessory organs i.e., salivary glands, liver, gallbladder, and pancreas. ACE2 is expressed in relatively high levels in duodenum, small and large intestines, rectum, as well as in gallbladder. Thus, following the consumption of contaminated food the virus likely reaches the stomach passively; the reported adverse effects in other accessory organs like liver or pancreas are probably the result of excessive inflammation during severe COVID-19. **c** Central (brain, spinal cord) and peripheral nervous system as an infection route of SARS-CoV-2; ACE2, neuropilin-1 (NRP1) and CD147 that reportedly potentiate virus infectivity into the central nervous system are shown. The molecular pathways involved in SARS-CoV-2 infection in human (e.g., lung) cells are depicted in Fig. 1

Interestingly, it has been shown in mice that SARS-CoV-1 (the coronavirus that caused the SARS epidemic in 2003) infection downregulates ACE2 protein (but not ACE) contributing to severe lung injury [23]. The ACE2-dependent pathogenicity of SARS-CoV-2 has been also confirmed in mice expressing human ACE2 [24]. Suppressed ACE2 expression and locally increased ANGII production can induce leakage of pulmonary blood vessels (a hallmark in ARDS pathogenesis) via AT1R stimulation [25]. Notably, in a model of lung injury being mediated by direct binding of nanoparticles to ACE2, which led to suppression of ACE2 expression levels and activity, administration of losartan (an AT1R antagonist) ameliorated nanoparticle-induced lung injury [26]. Likewise, extensive lung infection by SARS-CoV-2 in COVID-19, triggers capillary leakage which if sustained may lead to viremia (i.e., the presence of infectious virus in the circulation), local over-activation of the ACE/ANGII/AT1R signaling due to ACE2 diminishment, extensive inflammation and the so-called “cytokine storm” (Fig. 2a). Worth mentioning is however, that the etiology of “cytokine storm” remains largely elusive and may be well triggered by mechanisms not directly related to ACE2 through modulation of pulmonary macrophages, dendritic cells and/or neutrophils [27–30]. The alarming “cytokine storm”-related pro-inflammatory signals spread throughout the body most likely triggering ACE2 overexpression and thus increased ACE2/ANG(1–7)/MASR signaling as a counterbalancing effect. Given the extensive expression of ACE2 in most human organs, which is now exaggerated because of the pro-inflammatory alarming cytokines, the potentially (in cases of viremia) circulating virus can attack most vital organs (e.g., kidneys and heart). This vicious cycle may then accelerate due to infection-related locally increased ANGII production, which exaggerates ACE/ANGII/AT1R signaling causing systemic failure. Consistently to this hypothesis, postmortem examination of patients with COVID-19 revealed the existence of SARS-CoV-2 in multiple (apart from the lung) organs including pharynx, heart, liver, brain, and kidneys further supporting the broad organotropism of the virus [31, 32]. Worth noting is, that although there is seemingly no detectable viremia during asymptomatic infection or in the absence of clinical disease [33], SARS-CoV-2 RNA has been detected in blood samples from patients with mild symptoms [34] and detectable viral RNA in blood is a strong prognostic factor for clinical deterioration [35].

In line with these notions, the enrichment of all SARS-CoV-2 infection-related cellular modules (i.e., ACE2, TMRSS2 and CTSB, CTSL) in the gastrointestinal tract [17, 36] explain diarrhea as a major symptom of COVID-19 and SARS-CoV-2 RNA isolation from stool [33, 37, 38] (Fig. 2b). Given that SARS-CoV-2 productively infects human gut enterocytes [36] or human intestinal organoids [39] it is plausible that human intestinal tract represents a major entry and replication site for SARS-CoV-2 due to consumption of contaminated food. In support, intra-gastric inoculation of SARS-CoV-2 in a mouse model expressing human ACE2 caused productive infection and most interestingly led to pulmonary pathological changes [40]. A significant association between liver dysfunction and mortality of COVID-19 patients has been also reported [41, 42], which may relate to direct viral infection (still questionable due to relatively low ACE2 expression levels in the liver [17]); to indirect damage because of drug-induced liver injury or because of COVID-19-triggered systemic inflammation [43]. Analyses of severe COVID-19-induced biochemical alterations in the liver have shown the elevation of liver enzymes, such as alanine aminotransferases and aspartate aminotransferases, and significantly lower albumin levels [43, 44] and thus, liver markers should be monitored continuously during COVID-19 evolvement. ACE2 and TMPRSS2 are highly expressed in gallbladder [17], whereas regarding pancreas ACE2 is expressed in exocrine tissue microvasculature and in a subset of pancreatic ducts with TMPRSS2 expression being restricted to ductal cells [45, 46]. Notably, both ACE2 and TMPRSS2 are rarely expressed in single pancreatic β cells from donors with or without diabetes [45, 46] suggesting that SARS-CoV-2 cannot directly infect β cells.

Similarly, COVID-19 impact to the nervous system may relate to SARS-CoV-2 infection-mediated systemic imbalance of the neuroprotective ACE2/ANG(1–7)/MASR axis signaling [47, 48] or to direct effects mediated by SARS-CoV-2 neurotropism. SARS-CoV-2 canonical cell entry factors i.e., ACE2 and TMPRSS2 are expressed in glial cells, neurons, endothelial and arterial smooth muscle cells in the brain [49], while neuropilin-11 or CD147 may also facilitate SARS-CoV-2 entry into the central nervous system [7, 9] (Fig. 2c). In support SARS-CoV-2 can directly target neurons of 3D human brain organoids [50, 51] and the virus has been found in cerebrospinal fluid and neuronal cells [49, 52] indicating that SARS-CoV-2 is neuroinvasive, neurotropic and neurovirulent. The two main infection pathways are likely the hematogenous and the neuronal, with the olfactory route (where nasal cell express high levels of ACE2 [18]), along with the lymphatic tissue and the cerebrospinal fluid likely playing a significant role in SARS-CoV-2 neuroinvasion [52]. Most common COVID-19 neurological symptoms are headache, dizziness, hypogeusia and hyposmia, with rarer being severe symptoms like acute cerebrovascular disease, meningitis/encephalitis, acute necrotizing hemorrhagic encephalopathy, or even acute Guillain–Barré syndrome [52]. SARS-Cov-2 can also affect neuronal function indirectly by extensive inflammation-mediated increase of circulating cytokines which can penetrate the damaged blood brain barrier [52].

Conclusively, regarding primary sites of SARS-CoV-2 infection although lungs (Fig. 2a) and likely the gastrointestinal tract (Fig. 2b) are grounds zero during the infection process, SARS-CoV-2 and/or COVID-19 also tear multiple organ systems, with major targets (because of high ACE2, TMPRSS2 expression) being the heart and kidneys.

We propose that (a) the imbalance in the action of ACE- and ACE2-derived peptides, i.e., the increased ANGII versus ANG(1–7) ratio, which occurs due to SARS-CoV-2 binding-mediated diminishment of ACE2 expression, along with, (b) the high ACE-2 expression levels-related increased tropism of the virus to vital human organs (e.g., kidneys and heart) are major drivers of COVID-19 pathobiology. Thus, those at high risk for severe COVID-19 (e.g., the elderly or those with underlying morbidities) should probably be (among others; see below) on prophylactic treatment with RAS inhibitors (e.g., AT1R antagonists or ACE inhibitors) to decrease systemic damage risk and thus blunt COVID-19-associated morbidity and mortality.

Given the aforementioned sequence of events, tissues affected and downstream pathologies, the design of COVID-19 therapeutics (until the discovery of an effective highly specific anti-viral drug and/or a vaccine) may be complex, but it also presents with several potentially druggable opportunities. Overall, it is now understood that acute COVID-19 is a two-phase disease, including (a) infection and spreading of the virus mainly in the respiratory and gastrointestinal tracts, and, (b) ARDS (which can occur after a temporal improvement) and the uncontrolled immune response of the host [53] which can then lead to worsening of ARDS, development of multi-organ pathologies and systemic failure (Fig. 2) [54]. Effective therapeutic treatments should thus probe both SARS-CoV-2 inhibition through better understanding of its life cycle and also the side-effects induced by COVID-19 due to immune system overactivation and organ dysfunction caused by the broad organotropism of SARS-CoV-2.

## Targeting the life cycle of the virus

For prophylactically targeting the virus life cycle (*phase 1* of the disease), the magic bullet will be the development of an effective vaccine which can induce SARS-CoV-2-specific neutralizing antibodies. Indeed, more than 90 vaccines are being developed against SARS-CoV-2 by researchers in companies and universities worldwide where research teams are trialing different technologies, some of which have not been used in a licensed vaccine before [55]. Most of these vaccines were found to induce protective neutralizing antibodies and CD8^+^ T cell responses to wild-type (D614) and D614G mutant SARS-CoV-2 in mice, rats, guinea pigs, rabbits, and non-human primates [56–62], while some of them are already being tested in advanced clinical trials with encouraging results [63–65] indicating a potential to provide protection against COVID-19 [66, 67]. Alternatively, the isolation of virus-specific human monoclonal antibodies exerting SARS-CoV-2 neutralization activity from memory B cells (e.g., by high-throughput single-cell sequencing) of SARS-CoV-2 infected and recovered individuals [68–74] or from genetically-humanized mice [73] can be potentially applied as prophylactic treatment in individuals at high risk for infection or as a post-exposure therapy to limit or treat severe disease. To this end noncompeting antibody cocktails (e.g., REGN10987/REGN10933 or S2E12/S2M11) that target nonoverlapping epitopes on the SARS-CoV-2 S protein can prevent the generation of escape S mutants [75] and are highly effective in both hamsters and rhesus macaques COVID-19 models [76, 77]; additional cocktail (or not) neutralizing antibody preparations were found to be protective in both mice and non-human primates COVID-19 models [78–80] as well as in an interim analysis of a phase 2 trial [81]. Worth mentioning is that beyond the potential prophylactic and/or therapeutic usage of SARS-CoV-2 neutralizing antibodies it would be essential to monitor SARS-CoV-2 seroprevalence and neutralizing activity in donors’ and patients’ blood during the on-going second wave of the COVID-19 pandemic, as well following the initiation of community vaccination; pseudovirus [82] or virus-, cell-free ([83], recently received FDA authorization for use) neutralization assays can be used in this screening.

In another approach, soluble ACE2 (e.g., rhACE2; APN01, GSK2586881), although with less affinity to the virus and half-life as compared to well-selected specific antibodies, can be used as a decoy to neutralize the virus due to competitive binding with cellular ACE2. Supportively, SARS-CoV-2 infections were suppressed in engineered human tissues by clinical-grade soluble human ACE2 [84]. SARS-CoV-2 direct binding to ACE2 can be also suppressed by antibodies or small molecules that target ACE2, such as SSAA09E2, which inhibits SARS-CoV-1 interaction with ACE2 [85]. To reduce the available ACE2 viral binding sites, approaches that suppress *ACE2* and/or *TMPRSS2* gene expression (e.g., TNF or androgen inhibitors) [17] may be employed. To test this hypothesis, it would be interesting to investigate e.g., whether prostate cancer patients on anti-androgens have less severe COVID-19 due to *TMPRSS2* gene suppression. Regarding *ACE2* and given the anticipated toxic effects of systemic loss-of ACE2/ANG(1–7)/MASR signaling, any relevant intervention should be transient and with close monitoring of the patients’ clinical condition. An alternative and probably safer, *versus* ACE2 inhibition, approach would be the use of TMPRSS2 inhibitors. TMPRSS2 is druggable and camostat mesylate partially blocked SARS-CoV-2 cell infection [4] while preliminary reports showed that it reduced the severity of COVID-19 sepsis [86]; thus, several clinical trials are currently ongoing to test whether camostat mesylate could be repurposed and utilized to combat the current pandemic [87]. TMPRSS2 inhibition could also reduce viral tropism at the initial site of SARS-CoV-2 infection and enhance anti-viral humoral immune responses of the host [88, 89]. Additional steps to be targeted at SARS-CoV-2 life cycle include its fusion with cell membrane as well as clathrin-mediated endocytosis. In these pathways, ikarugamycin (clathrin-mediated endocytosis inhibitor; [90]), dynasore or its analogs (dynamin inhibitor; [91]), as well as latrunculin B (actin depolymerizing drug, [92]) can be tested in cell-based or preclinical models. Additionally, the small molecule SSAA09E3 was found to suppress the fusion of the host cellular membrane with the virus membrane in a SARS-CoV-1 infection model [85]. Other fusion inhibitors that can be considered include 25-hydrocholesterol which showed broad anti-coronavirus activity by blocking membrane fusion and inhibiting SARS-CoV-2 infection in lung epithelial cells and viral entry in human lung organoids [93].

In the cell, novel SARS-CoV-2-specific antiviral drugs can target the virus’ main protease (Mpro) due to its critical role in processing the polyproteins that are translated from the viral RNA [94, 95] or the SARS-CoV-2 RNA-dependent RNA polymerase (RdRp) that is used for viral genome replication and transcription of viral genes [96]. As a temporary alternative, existing antiviral drugs can be tested (repurposing) since according to molecular docking studies they bind tightly to SARS-CoV-2 RdRp [97]. Here, various anti-polymerase drugs that have been approved for use against other viruses are currently tested and Remdesivir (an RdRp inhibitor) [2] has shown promising clinical effects in patients with severe COVID-19 [98, 99] and in October 22 it became the first COVID-19 drug approved by FDA; worth mentioning, however, is that some well-designed studies, including the WHO’s giant Solidarity trial, challenge its therapeutic value [100]. In general, as was found with other viral pathogens, targeting either Mpro or RdRp are currently the most promising anti-SARS-CoV-2 approaches. Additional intracellular modules of the virus life cycle that can be targeted include tubulin and/or CTSB/L (Fig. 1). Colchicine is an efficient inhibitor of tubulin polymerization [101] and thus of virus loaded endosomes and could be a promising treatment in inhibiting the early phases of virus infection. Similarly, the lysosomotropic compounds chloroquine or hydroxychloroquine inhibit (non-specifically) the activity of endosomal/lysosomal compartments likely reducing the initial phases of viral infection; also, by suppressing lysosomal activity these compounds may also suppress renin production [17] further relaxing the potentially harmful ACE/ANGII/AT1R overactive signaling. Both chloroquine and hydroxychloroquine have been already used as therapeutics against COVID-19 [102]. Yet, given their cardiotoxic effects [103] they should probably be used only in the context of clinical trials or monitored conditions, and likely in early phases of the infection. Indeed, the RECOVERY trial announced the ending of its hydroxychloroquine arm, as it was concluded that the drug had no clinical benefit for hospitalized patients with COVID-19 [104], while in an open label, randomized controlled trial, adverse events were higher in hydroxychloroquine recipients than in non-recipients [105]. Several existing CTSB/L specific inhibitors, including intracellular regulators (e.g., cystatins) of cathepsin B activity [106], can be also considered. Notably, cathepsins B/L activity is upregulated during aging [107] and has been correlated with atherosclerotic vascular disease and arterial stiffening [108]; these findings further support the notion that age and cardiovascular morbidities are main risk factors for COVID-19. At the dark side of inhibiting the acidic cellular endosomal/lysosomal compartments or CTSB/L is their functional involvement in MHC class II antigen presentation (Fig. 1) [109]. It can be assumed that the usage of chloroquine/hydroxychloroquine; of CTSB/L specific inhibitors or even the excessive loading of the endosomal compartments by the virus could reduce MHC class II antigen presentation. Suppressed antigen presentation along with the presence of O-linked glycans at the surface of the virus [110] could result in evasion of the immune system. Thus, virus clearance may proceed via proteasome-mediated MHC class I-related antigen cross presentation [111] and the activity of MHC-I dependent cytotoxic T immune cells. Proteasome activity is downregulated during aging [112] and also proteasome is less responsive to IFN-γ in senescent cells [113], which would then result in reduced antigen presentation by MHC class I molecules and therefore reduced immune responses in aged patients. In this context, small molecules and/or drugs that activate proteasome and in parallel suppress cathepsins B/L activity could provide useful therapeutics against COVID-19. Finally, the finding that SARS-CoV-2 infection reshapes essential cellular pathways, such as nucleic acid metabolism, splicing, translation and carbon metabolism in human cells [114] indicates that a number of small molecule inhibitors that target these pathways could contribute to preventing viral replication in human cells.

## Targeting the adverse effects of COVID-19

In relation to *phase 2* of the disease, i.e., ARDS induction and the alarming “cytokine storm” (Fig. 2a) which can then lead to systemic failure (see above), the ANG(1–7) peptide (or non-peptide analogs) can likely rescue local SARS-CoV-2 infection-related ACE2 loss and the subsequent ANGII accumulation, leading to reactivation of the anti-inflammatory MASR signaling pathway (Fig. 1). Consistently, ANG(1–7) protects endothelial cells from inflammation and high glucose-mediated injury [115], enhances insulin action [116] and was protective in heart failure [117] and stroke [118]. Because of SARS-CoV-2 infection-induced overactivation of the pro-oxidative ACE/ANGII/AT1R axis, which can trigger endothelial dysfunction due to unbalanced reactive oxygen species and nitric oxide ratios in the vessel wall [119], the use of drugs (or small molecules) that activate anti-oxidant cellular defenses [e.g., the Nuclear factor erythroid 2-related factor 2 (Nrf2), pathway] or act as radical scavengers could be an additional prophylactic intervention. Reportedly, COVID-19 may also cause endotheliitis in several organs as a direct consequence of both viral involvement and of the host inflammatory response [120]. Also, COVID-19 correlates with venous thromboembolism and disseminated intravascular coagulation [121, 122]; in this context anticoagulant medications can be used as either prophylactic and/or therapeutic treatment.

Finally, regarding COVID-19-induced “cytokine storm”, i.e., the uncontrolled systemic inflammatory response that relates to the release of high amounts of pro-inflammatory cytokines along with complement components, coagulation dysfunction and immunological “misfiring” [68, 123–126]; the idea of adjunct immunotherapies which inhibit key pro-inflammatory pathways such as IL-6 signaling [127, 128] is a reasonable approach. More specifically, studies in animal models and cell-based assays following SARS-CoV-2 infection, as well as serum and transcriptional profiling of COVID-19 patients, revealed an exaggerated abnormal inflammatory response being marked by reduced levels of type I and III IFNs, along with increased chemokines and IL-6 expression [129]. Also, a single-cell atlas of immune responses in patients with severe COVID-19 revealed a reconfiguration of peripheral immune cells phenotype during life-threatening COVID-19, including HLA class II downregulation, a heterogeneous IFN-stimulated gene signature and a developing neutrophil population that relates to plasmablasts which appear in patients developing ARDS and requiring mechanical ventilation [130]. Interestingly, patients with life-threatening COVID-19 pneumonia (but not with asymptomatic or mild SARS-CoV-2 infection) had neutralizing IgG auto-Abs against IFNs [131] or errors of TLR3- and IRF7-dependent type I IFN immunity [132] suggesting that inborn errors of type I IFN immunity underlies severe COVID-19. Across these lines of research it was found that coordinated CD4^+^, CD8^+^ T cells and antibody responses are protective, whereas uncoordinated responses frequently fail to control disease, with a connection between aging and impaired adaptive immune responses to SARS-CoV-2 [133]. Furthermore, lymphocytopenia (especially reduced CD8^+^ and CD4^+^ T cell counts upon admission), was predictive of disease progression and correlated with high levels of IL-8 and IL-6 also in patients with severe or critical disease [134]. Interestingly, disease severity seems to mostly stem from host factors such as lymphocytopenia, the associated “cytokine storm” and age, whereas genetic variation of the virus was not shown to associate with patients’ clinical outcome [134]. Nonetheless, an excessive inhibition of the immune system by corticosteroids should be avoided since dampening of a cytokine response could allow excessive viral replication. In support, the UK RECOVERY trial found that dexamethasone (a common steroid) could reduce COVID-19 fatalities by as much as one-third when administered to patients who require supplemental oxygen or are on ventilators (RECOVERY); however, dexamethasone treatment has not been shown to offer a benefit for people with mild COVID-19 who do not need oxygen support, possibly because it weakens defenses against the virus itself [135]. Moreover, the observations that IFNs induce *ACE2* gene expression [17, 19] prompts for an urgent detailed analysis of how key effectors of the immune system regulate the *ACE2*, *TMPRSS2* and *CTSB/L* genes and hence, tropism and infection rates of the virus in targeted human tissues.

Interestingly, it has been recently reported the existence of SARS-CoV-2-specific T cells in individuals with no history of SARS, COVID-19 or contact with individuals who had SARS and/or COVID-19; these T cells target (among others) SARS-CoV-2 N protein [136]. Consistently, it was found that S-reactive CD4^+^ T cells in SARS-CoV-2-unexposed healthy donors reacted primarily to C-terminal S protein epitopes, which show a higher homology to spike glycoproteins of human endemic coronaviruses versus N-terminal epitopes. Moreover, S-reactive T cell lines that were generated from SARS-CoV-2-naive SARS-CoV-2-unexposed healthy donors were found to respond similarly to the S protein (C-terminus) of the human endemic coronaviruses OC43 and 229E and, interestingly enough, of SARS-CoV-2, demonstrating the likely presence of S-cross-reactive T cells, probably generated during past infections with endemic coronaviruses [137]. The presence of S protein cross-reactive T cells in a significant portion of the general population is of critical importance as apart from affecting the dynamics of the current pandemic it may also have important implications for the design and analysis of upcoming COVID-19 vaccine trials.

Towards the development of an effective vaccine, it is encouraging that SARS-CoV-2 is likely mutating slowly with most variants with amino acid changes at RBD being less infectious [138]. Nonetheless, variants which impact on virus infectivity such as the D614G mutation which alters SARS-CoV-2 fitness by increasing its replication ex vivo and transmission in vivo [139–141] or others (e.g., variants A475V, L452R, V483A, F490L) that affect reactivity to neutralizing antibodies and sera from convalescent patients [141, 142] have emerged. Furthermore, it was found that the majority of glycosylation deletions were less infectious, whereas deletion of both N331 and N343 glycosylation drastically reduced infectivity, further supporting the importance of glycosylation for viral infectivity; notably, N234Q was markedly resistant to neutralizing antibodies, whereas N165Q became more sensitive [142]. Thus, a close monitoring of novel SARS-CoV-2 variants with a possible fitness advantage is needed.

## Conclusions

COVID-19 is a two-phase disease being marked by (*phase 1*) rapid virus propagation due to the wide expression of *ACE2*, *TMPRSS2* and *CTSB/L* genes (along with the other putative alternative receptors and/or attachment factors) in tissues of the respiratory and gastrointestinal tract, as well as by (*phase 2*) host- [143], and probably sex- [144] and/or age-specific [145, 146] uncontrolled inflammatory immune responses which drive aggressive inflammation, hyper-cytokinemia, and (due to the broad organotropism of SARS-CoV-2) collateral tissue damage and systemic failure because of imbalanced ACE/ANGII/AT1R and ACE2/ANG(1–7)/MASR axes signaling. Discussed notions provide a basis of resources for (*a*) future investigations of COVID-19 pathogenesis and (*b*) possible combinatorial therapeutic approaches (Table 1); lists of non-FDA and FDA approved potential treatments targeting specific mechanisms that likely mediate COVID-19 complications are reported in Tables 2, 3 respectively.

**Table 1:** Possible targets to alleviate the life-threatening complications of COVID-19

*Pre-Phase 1*
- Vaccine (e.g., against the SARS-CoV-2 S protein) ***1*** (for the various technologies employed see also text and [149–152])
*Phase 1 of the disease: Life cycle of the virus (extracellular – early steps of infection)*
- SARS-CoV-2 neutralizing monoclonal antibodies ***1*** (see text and [153–155]; because antibody-dependent enhancement of disease [156] cannot be reliably predicted after either vaccination or treatment with antibodies, the on-going clinical trials for COVID-19 immune interventions should depend on careful analyses for safety in humans; also, preferentially the development of neutralizing antibodies after vaccination should be monitored) – (neutralizing antibodies from Eli Lilly and Regeneron Pharmaceuticals Inc. have received FDA emergency use authorization and GlaxoSmithKline/Vir Biotechnology has moved an anti-SARS-CoV-2 mAb into Phase 3 clinical trials) - Soluble ACE2 (decoy for virus) ***2*** (a recent development is this field is the production of engineered human ACE2 with optimum binding to the S protein of SARS-CoV-2 [157])
- Antibodies or small molecules that target ACE2 ***2***
- Treatments that suppress *ACE2* and/or *TMPRSS2* genes expression ***2***
- TMRPSS2 protease inhibitors ***3***
- Inhibitors of membrane fusion and/or clathrin-mediated endocytosis ***4***
*Phase 1 of the disease: Life cycle of the virus (intracellular)*
- Tubulin polymerization inhibitors ***4***,4*,***7***
- Inhibitors of the endosomal/lysosomal compartments 4,4* (recent studies in non-human primates do not support the use of hydroxychloroquine -either alone or in combination with azithromycin- for the treatment of COVID-19 in humans [158]; also, chloroquine was not found to inhibit infection of human lung cells with SARS-CoV-2 [159])
- CTSB/L specific inhibitors ***4***
- Small molecule inhibitors of cellular pathways reshaped by SARS-CoV-2 infection (not shown)
- Inhibitors of the virus’ main protease ***5***,***6***
- Virus’ RNA-dependent RNA polymerase inhibitors ***5***,***6***
- MHC class II/MHC class I antigen presentation enhancement ***8***
*Phase 2 of the disease: adverse effects of COVID-19*
- ACE inhibitors, AT1R blockers ***10***–***12***
- The ANG(1–7) peptide (or non-peptide analogs) ***10***–***12***
- Antioxidants or radical scavengers ***10***–***12***
- Adjunct immunotherapies (or corticosteroids) to mitigate “cytokine storm” (e.g., inhibition of IL-6 signaling) ***10***–***12*** (notably, the use of hydrocortisone [160] or dexamethasone [161] showed some beneficial effects on mortality, organ support, days alive and free of mechanical ventilation in patients with severe COVID-19)
- Anticoagulant medications to alleviate intravascular coagulation (not shown)
- Additional life-supporting measures (e.g., ventilation or intubation) (not shown)

Numbers in bold italics (see, respective red color numbers in Figs. 1, 2) indicate major components in SARS-CoV-2 life cycle and in COVID-19 progression and pathology

**Table 2:** Treatments (non-FDA approved) which can potentially suppress SARS-CoV-2 infection rates and/or COVID-19 complications (see also, Table 1)

Intervention	Study type	Biologic efficacy	References
*Induction of SARS-CoV-2-specific neutralizing antibodies*			
Recombinant Novel Coronavirus Vaccine Gam-COVID-Vac Vaccine	Phase 3 clinical trial (viral two-vector vaccine based on the human adenovirus fused with the S protein of SARS-CoV-2)	Unknown	[162, 163]
Adsorbed COVID-19 (inactivated) Vaccine SARS-CoV-2 Vaccine (Vero cell)	Phase 3 clinical trial (absorbed inactivated SARS-CoV-2)	Unknown	[164, 165]
mRNA-1273 vaccine	Phase 3 clinical trial (mRNA-based vaccine that encodes for a full-length, prefusion stabilized S protein of SARS-CoV-2)	Unknown	[166]
*SARS-CoV-2 neutralizing monoclonal antibodies*			
COV2-2196, COV2-2130	In vitro and in vivo study (mouse)	Reduce viral burden and level of inflammation in mouse’s lungs	see text
P2C-1F11, P2B-2F6, P2C-1A3	In vitro (antibodies derived from 8 individuals infected with SARS-CoV-2)	Substantial neutralizing activities against SARS-CoV-2 infection	see text
CB6	In vivo (specific human antibodies administrated in rhesus macaques)	Prophylactic group: prevention of SARS-CoV-2 infection. Treatment group: reduced SARS-CoV-2 titre	see text
*Soluble angiotensin converting enzyme 2 (ACE2) (decoy for virus)*			
GSK2586881	Phase 2 clinical trial (recombinant human ACE2 in ventilated patients with ARDS)	Unknown	[167]
RhACE2 APN01	Ongoing phase 2 clinical trial (recombinant human ACE2)	Unknown	[168]
*Antibodies or small molecules that target ACE2*			
SSAA09E2	In vitro (small molecule added to 293 T and Vero cells)	Inhibits fusion of the SARS-S envelope with the host cellular membrane	see text
COV2-2196 COV2-2381	In vivo (monoclonal antibodies administrated in rhesus macaques)	Prophylactic group: prevention of SARS-CoV-2 infection	see text
*TMRPSS2 protease inhibitors*			
Camostat mesylate	In vitro (lung cell line)	Blocks SARS-CoV-2 infection of lung cells	see text
*Inhibitors of membrane fusion and/or clathrin-mediated endocytosis*			
Ikarugamycin	In vitro (H1299 cells)	Acutely inhibits clathrin‐mediated endocytosis (CME)	see text
Dynasore, Dyngo 4a, Dyngo 6a	In vitro	Inhibit specifically dynamin and clathrin-mediated endocytosis	see text
Latrunculin b	In vitro	Inhibits Australian bat lyssavirus G-mediated entry into HEK293T cells through actin depolymerization	see text
SSAA09E3	In vitro (small molecule added to 293 T and Vero cells)	Prevents fusion of the SARS-CoV-2 membrane with the host cellular membrane	see text
*Virus’ RNA-dependent RNA polymerase (RdRp) inhibitors*			
Setrobuvir, IDX-184, YAK	In vitro	Bind to RdRp tightly and hence may contradict the polymerase function	see text
*Cathepsin L inhibitors*			
SSAA09E1, Oxocarbazate, MDL-28170, K11777, EST	In vitro (293 T cells)	Blocks SARS CoV-2 entry	see text, [169]
*Inhibitors of cellular pathways reshaped by SARS-CoV-2 infection*			
Cycloheximide	In vitro (human Caco2 cells)	Inhibits translation elongation and SARS-CoV-2 replication	see text
Emetine	In vitro (human Caco2 cells)	Inhibits the 40S ribosomal protein S14 and SARS-CoV-2 replication	see text
Pladienolide B	In vitro (human Caco2 cells)	Inhibits splicing factor SF3B117 and SARS-CoV-2 replication	see text
2-Deoxy-d-glucose	In vitro (human Caco2 cells)	Blocks glycolysis and inhibits SARS-CoV-2 replication	see text
Ribavirin	In vitro (human Caco2 cells)	Inhibits inosine monophosphate dehydrogenase and SARS-CoV-2 replication	see text
NMS-873	In vitro (human Caco2 cells)	Inhibits the AAA ATPase p97 and SARS-CoV-2 replication	see text
ANG(1–7) peptide			
Angiotensin 1–7, TXA127	Ongoing Phase 3 clinical trial	Unknown	[170, 171]

**Table 3:** FDA approved drugs/therapies which can likely target SARS-CoV-2 life cycle and/or COVID-19 complications (see also, Table 1)

Intervention	Drug category	Approved indication	Biologic and/or clinical efficacy	Serious adverse events	References
*Suppression of ACE2 gene expression*					
Dutasteride	5-a reductase inhibitors	Benign prostate hyperplasia	Reduces ACE2 and inhibits internalization of SARS-Cov-2 S protein in vitro	None reported	[172, 173]
Anti-TNF agents	Monoclonal antibodies	Autoimmune diseases	Lower incidence of COVID-19 disease in patients with Inflammatory Bowel Disease (Retrospective cohort study and SECURE-IBD database)	Serious infections, demyelinating disorders, drug-induced lupus, may increase risk of malignancies	[174–176]
*Suppression of TMPRSS2 gene expression*					
Anti-TNF agents	Monoclonal antibodies	Autoimmune diseases	Lower incidence of COVID-19 disease in patients with Inflammatory Bowel Disease (Retrospective cohort study and SECURE-IBD database)	Serious infections, demyelinating disorders, drug-induced lupus, may increase risk of malignancies	[174–176]
Homoharringtonine (Omacetaxine)	Protein translation inhibitor	Chronic myeloid leukemia	Reduces SARS-CoV-2 pseudoviral entry, in vitro	Myelosuppression	[177–180]
Verteporfin	Photosensitizer for photodynamic therapy	Subfoveal choroidal neovascularisation	Reduces SARS-CoV-2 pseudoviral entry, in vitro	Visual disturbances	[177–180]
Cilnidipine	Calcium channel blocker	Hypertension	Reduces SARS-CoV-2 pseudoviral entry, in vitro	None reported	[177–180]
Dasatinib	Tyrosine kinase inhibitor	Chronic myeloid leukemia, acute lymphoblastic leukemia	Reduces SARS-CoV-2 pseudoviral entry, in vitro	Cytopenia, pleural effusion	[177–180]
Venetoclax	Selective BCL-2 inhibitor	Chronic lymphocytic leukemia, small lymphocytic lymphoma	Reduces SARS-CoV-2 pseudoviral entry, in vitro	Neutropenia, lymphopenia, reactivation of hepatitis B, interaction with CYP3A inhibitors and azithromycin	[177–180]
*Inhibition of TMRPSS2 protease*					
Nafamostat mesylate	Serine protease inhibitor	Pancreatitis, anticoagulant during extracorporeal circulation supportive treatment	Inhibits SARS-CoV-2 S-mediated entry into host cells, in vitro	Bleeding	[181, 182]
*Inhibition of clathrin-mediated endocytosis*					
Umifenovir	Antiviral	Influenza A and B	Its combination with lopinavir/ritonavir ends to better outcome in COVID-19 patients *versus* only lopinavir/ritonavir	Hepatotoxicity in combination with lopinavir/ritonavir	[183, 184]
Chlorpromazine	Antipsychotic	Schizophrenia, bipolar disorders	In vitro inhibition of viral replication of coronaviruses	Parkinsonism, hypotension	[185, 186]
*Inhibition of virus’ main protease*					
Glecaprevir	Antiviral	Hepatitis C	Binds with high affinity to SARS-CoV-2 main protease and inhibits it, in vitro	None reported	[187]
Maraviroc	Antiviral	Human Immunodeficiency Virus	Binds with high affinity to SARS-CoV-2 main protease and inhibits it, in vitro	None reported	[187]
*Inhibition of virus’ RNA-dependent RNA polymerase (RdRp)*					
Ribavirin	Antiviral	Respiratory Syncytial Virus infection, Hepatitis C, some viral hemorrhagic fevers	Bind to the SARS-CoV-2 RdRp tightly in vitro, suppressing its function	Neutropenia, thrombocytopenia, neuropsychiatric toxicity	see text, [188]
Remdesivir	Antiviral	Broad-spectrum antiviral medication	Bind to the SARS-CoV-2 RdRp tightly in vitro, contradicting its function	Hepatotoxicity, nephrotoxicity	see text, [184]
Sofosbuvir	Antiviral	Hepatitis C	Bind to the SARS-CoV-2 RdRp tightly in vitro, contradicting its function	None reported	see text
Galidesivir	Antiviral	Hepatitis C	Bind to the SARS-CoV-2 RdRp tightly in vitro, contradicting its function	None reported	see text, [184]
Tenofovir	Antiviral	Hepatitis B, Human Immunodeficiency Virus	Bind to the SARS-CoV-2 RdRp tightly in vitro, contradicting its function	Renal and bone toxicity	see text, [189]
Favipiravir	Antiviral	Influenza	Increases clinical recovery over 7 days and reduces fever, cough, and respiratory problems in COVID-19 patients	Teratogenicity, embryotoxicity	[190]
*Inhibition of tubulin polymerization*					
Colchicine	Anti-inflammatory	Gout, rheumatic diseases, pericarditis	Improves time to clinical deterioration in COVID-19 patients	Diarrhea	see text, [191]
*Inhibition of the endosomal/lysosomal compartments*					
Chloroquine Hydroxychloroquine	Anti-malarial	Malaria, lupus erythematosus, rheumatoid arthritis	COVID-19 load reduction/disappearance with hydroxychloroquine	Cardiac arrest, retinotoxicity	see text, [184, 192]
*Cathepsin L inhibitor*					
Teicoplanin	Antibacterial	Gram positive bacteria (methicillin-resistant *Staphylococcus aureus*, *Enterococcus faecalis*)	Inhibits SARS Cov-2 replication cycle in vitro	None reported	[190, 193]
*Antioxidants*					
Thymoquinone Egcg Vit D3	Nutritional supplements	Oxidative stress, vitamin deficiency	Combination of the 3 compounds may prevent and/or decrease SARS-CoV-2 infection severity through activation of Nrf2 transcription factor	None reported	[194]
Zinc	Nutritional supplement	Oxidative stress, zinc deficiency	Clinical improvement in COVID-19 patients	None reported	[195, 196]
*Free radical scavenger*					
Ergothioneine	Nutritional supplement	Oxidative stress	Potential reduction of severity and mortality of COVID-19 disease	None reported	[197]
*Immunotherapies to mitigate “cytokine storm”*					
Tocilizumab	IL-6 receptor blocking monoclonal antibody	Connective tissue diseases	Rapid improvement in clinical and laboratory measures of hyperinflammation in hospitalized patients with COVID-19 Reduce the risk of invasive mechanical ventilation or death in patients with severe COVID-19 pneumonia	Neutropenia, thrombocytopenia	[198]
Sarilumab	IL-6 receptor blocking monoclonal antibody	Rheumatoid arthritis	Rapid improvement in respiratory function and normalization of inflammatory markers	Neutropenia, thrombocytopenia, upper respiratory and urinary tract infection	[199]
Dexamethasone	Corticosteroid	Variety of medical uses	Increase in the number of ventilator-free days in COVID-19 patients with ARDS	Acne, insomnia, vertigo, increased appetite, weight gain, depression	[161]
*Angiotensin converting enzyme (ACE) inhibitors and angiotensin II type 1 receptor (AT2R1) blockers*					
ACE inhibitors	Anti-hypertensives	Hypertension	Reduce risk of 28-day death among hospitalized COVID-19 patients with coexisting hypertension and coronary artery disease Decrease the mortality of COVID-19	Angioedema, anemia	[200–203]
AT1R blockers	Anti-hypertensives	Hypertension	Decrease mortality in COVID-19 patients with hypertension	Angioneurotic edema, anemia, liver damage, renal failure, aggravation of angina and migraine	[200–203]

It is suggested that the evidence-based (i.e., from both pre-clinical models and clinical trials) use of specific therapeutic approaches/drugs that target modules in pathways ***1***–***12*** (Table 1) shown in Figs. 1 and 2, can provide possible means to alleviate the life-threatening complications of COVID-19. In addition, the emerged issues of convalescent plasma treatment effectiveness in severe COVID-19 patients [147]; the duration of anti-SARS-CoV-2 antibodies persistence in mild or severe COVID-19 recovered patients [148] and the possibility of auto-antibodies development against type I IFNs [131] or inborn errors of type I IFN immunity [132] in patients with life-threatening COVID-19 should be further investigated and addressed in adequately powered, randomized controlled trials. Finally, given the fact that COVID-19 has certainly an age-related component [145, 146], as clinical complications mostly develop in the elderly and in patients with non-communicable (age-related) diseases, additional efforts should also focus in those pathways that reportedly become dysfunctional during aging (e.g., reduced proteasome functionality that causes minimized viral MHC antigen presentation, immune senescence, age-related increased inflammation, unbalanced ACE/ANGII/AT1R and ACE2/ANG(1–7)/MASR regulatory axes, etc.) and likely exaggerate the clinical complications of severe COVID-19.

## Data Availability

Not applicable.

## Abbreviations

ACE: Angiotensin converting enzyme
ACE2: Angiotensin converting enzyme 2
AGT: Angiotensinogen
ANGII: Angiotensin II
ARDS: Acute respiratory distress syndrome
AT2R1: ANGII type 1 receptor
ATR2: ANGII type 2 receptor
COVID-19: Coronavirus disease 2019
CTSB: Cathepsin B
CTSL: Cathepsin L
HIV: Human immunodeficiency virus
IFN: Interferon
MASR: Vasodilation-promoting receptor MAS
Mpro: Virus’ main protease
NRP1: Neuropilin-1
RAS: Renin-angiotensin system
RdRp: RNA-dependent RNA polymerase
RBD: Receptor binding domain
S: Spike protein
SARS-CoV-1: Severe acute respiratory syndrome coronavirus 1
SARS-CoV-2: Severe acute respiratory syndrome coronavirus 2
TMPRSS2: Transmembrane serine protease 2
TNF: Tumor necrosis factor

## References

[1] Huang C, Wang Y, Li X (2020). Clinical features of patients infected with 2019 novel coronavirus in Wuhan, China. Lancet.

[2] Gao Y, Yan L, Huang Y (2020). Structure of the RNA-dependent RNA polymerase from COVID-19 virus. Science.

[3] Mahase E (2020). Covid-19: what treatments are being investigated?. BMJ.

[4] Hoffmann M, Kleine-Weber H, Schroeder S (2020). SARS-CoV-2 cell entry depends on ACE2 and TMPRSS2 and is blocked by a clinically proven protease inhibitor. Cell.

[5] Lan J, Ge J, Yu J (2020). Structure of the SARS-CoV-2 spike receptor-binding domain bound to the ACE2 receptor. Nature.

[6] Hoffmann M, Kleine-Weber H, Pöhlmann SA (2020). Multibasic cleavage site in the spike protein of SARS-CoV-2 is essential for infection of human lung cells. Mol Cell.

[7] Cantuti-Castelvetri L, Ojha R, Pedro LD (2020). Neuropilin-1 facilitates SARS-CoV-2 cell entry and provides a possible pathway into the central nervous system. BioRxiv..

[8] Cuervo NZ, Grandvaux N (2020). ACE2: evidence of role as entry receptor for SARS-CoV-2 and implications in comorbidities. Elife..

[9] Qiao J, Li W, Bao J, Peng Q, Wen D, Wang J, Sun B. The expression of SARS-CoV-2 receptor ACE2 and CD147, and protease TMPRSS2 in human and mouse brain cells and mouse brain tissues. Biochem Biophys Res Commun. 2020;S0006–291X(20)31783–6. 10.1016/j.bbrc.2020.09.042 PMC7489930 33008593

[10] Kuba K, Imai Y, Penninger JM (2006). Angiotensin-converting enzyme 2 in lung diseases. Curr Opin Pharmacol.

[11] Bindom SM, Lazartigues E (2009). The sweeter side of ACE2: physiological evidence for a role in diabetes. Mol Cell Endocrinol.

[12] Imai Y, Kuba K, Ohto-Nakanishi T, Penninger JM (2010). Angiotensin-converting enzyme 2 (ACE2) in disease pathogenesis. Circ J.

[13] Donoghue M, Hsieh F, Baronas E (2000). A novel angiotensin-converting enzyme-related carboxypeptidase (ACE2) converts angiotensin I to angiotensin 1–9. Circ Res.

[14] Tipnis SR, Hooper NM, Hyde R, Karran E, Christie G, Turner AJ (2000). A human homolog of angiotensin-converting enzyme. Cloning and functional expression as a captopril-insensitive carboxypeptidase. J Biol Chem.

[15] Li XC, Zhang J, Zhuo JL (2017). The vasoprotective axes of the renin-angiotensin system: physiological relevance and therapeutic implications in cardiovascular, hypertensive and kidney diseases. Pharmacol Res.

[16] Hashimoto T, Perlot T, Rehman A (2012). ACE2 links amino acid malnutrition to microbial ecology and intestinal inflammation. Nature.

[17] Gkogkou E, Barnasas G, Vougas K, Trougakos IP (2020). Expression profiling meta-analysis of ACE2 and TMPRSS2, the putative anti-inflammatory receptor and priming protease of SARS-Cov-2 in human cells, and identification of putative modulators. Redox Biol.

[18] Sungnak W, Huang N, Bécavin C (2020). SARS-CoV-2 entry factors are highly expressed in nasal epithelial cells together with innate immune genes. Nat Med.

[19] Ziegler CGK, Allon SJ (2020). SARS-CoV-2 receptor ACE2 is an interferon-stimulated gene in human airway epithelial cells and is detected in specific cell subsets across tissues. Cell.

[20] Tang D, Comish P, Kang R (2020). The hallmarks of COVID-19 disease. PLoS Pathog.

[21] Marin GH (2020). Facts and reflections on COVID-19 and anti-hypertensives drugs. Drug Discov Ther.

[22] Mancia G, Rea F, Ludergnani M, Apolone G, Corrao G (2020). Renin-angiotensin-aldosterone system blockers and the risk of COVID-19. N Engl J Med.

[23] Kuba K, Imai Y, Rao S (2005). A crucial role of angiotensin converting enzyme 2 (ACE2) in SARS coronavirus-induced lung injury. Nat Med.

[24] Bao L, Deng W, Huang B (2020). The pathogenicity of SARS-CoV-2 in hACE2 transgenic mice. Nature.

[25] Imai Y, Kuba K, Rao S (2005). Angiotensin-converting enzyme 2 protects from severe acute lung failure. Nature.

[26] Sun Y, Guo F, Zou Z (2015). Cationic nanoparticles directly bind angiotensin-converting enzyme 2 and induce acute lung injury in mice. Part Fibre Toxicol.

[27] Wong CK, Lam CW, Wu AK (2004). Plasma inflammatory cytokines and chemokines in severe acute respiratory syndrome. Clin Exp Immunol.

[28] Yoshikawa T, Hill T, Li K, Peters CJ, Tseng CT (2009). Severe acute respiratory syndrome (SARS) coronavirus-induced lung epithelial cytokines exacerbate SARS pathogenesis by modulating intrinsic functions of monocyte-derived macrophages and dendritic cells. J Virol.

[29] Herold S, Becker C, Ridge KM, Budinger GR (2015). Influenza virus-induced lung injury: pathogenesis and implications for treatment. Eur Respir J.

[30] Channappanavar R, Fehr AR, Vijay R, Mack M, Zhao J, Meyerholz DK, Perlman S (2016). Dysregulated type I interferon and inflammatory monocyte-macrophage responses cause lethal pneumonia in SARS-CoV-infected mice. Cell Host Microbe.

[31] Puelles VG, Lütgehetmann Μ (2020). Multiorgan and renal tropism of SARS-CoV-2. N Engl J Med.

[32] Su H, Yang M (2020). Renal histopathological analysis of 26 postmortem findings of patients with COVID-19 in China. Kidney Int.

[33] Wölfel R, Corman VM, Guggemos W (2020). Virological assessment of hospitalized patients with COVID-2019. Nature.

[34] Chang L, Zhao L, Gong H, Wang L, Wang L (2020). Severe acute respiratory syndrome coronavirus 2 RNA detected in blood donations. Emerg Infect Dis.

[35] Chen W, Lan Y, Yuan X (2020). Detectable 2019-nCoV viral RNA in blood is a strong indicator for the further clinical severity. Emerg Microbes Infect.

[36] Lamers MM, Beumer J, van der Vaart J (2020). SARS-CoV-2 productively infects human gut enterocytes. Science.

[37] Buscarini E, Manfredi G (2020). GI symptoms as early signs of COVID-19 in hospitalised Italian patients. Gut.

[38] Wong SH, Lui RN, Sung JJ (2020). Covid-19 and the digestive system. J Gastroenterol Hepatol.

[39] Zhou J, Li C (2020). Infection of bat and human intestinal organoids by SARS-CoV-2. Nat Med.

[40] Sun SH, Chen Q, Gu HJ (2020). A mouse model of SARS-CoV-2 infection and pathogenesis. Cell Host Microbe.

[41] Wu ZH, Yang DL (2020). A meta-analysis of the impact of COVID-19 on liver dysfunction. Eur J Med Res..

[42] Sharma A, Jaiswal P, Kerakhan Y (2020). Liver disease and outcomes among COVID-19 hospitalized patients—a systematic review and meta-analysis. Ann Hepatol.

[43] Zhong P, Xu J, Yang D (2020). COVID-19-associated gastrointestinal and liver injury: clinical features and potential mechanisms. Signal Transduct Target Ther..

[44] Abdulla S, Hussain A, Azim D, Abduallah EH, Elawamy H, Nasim S, Kumar S, Naveed H (2020). COVID-19-induced hepatic injury: a systematic review and meta-analysis. Cureus.

[45] Coate KC, Cha J, Shrestha S (2020). SARS-CoV-2 cell entry factors ACE2 and TMPRSS2 are expressed in the microvasculature and ducts of human pancreas but are not enriched in β cells. Cell Metab..

[46] Kusmartseva I, Wu W, Syed F (2020). Expression of SARS-CoV-2 entry factors in the pancreas of normal organ donors and individuals with COVID-19. Cell Metab..

[47] Sumners C, Horiuchi M, Widdop RE, McCarthy C, Unger T, Steckelings UM (2013). Protective arms of the renin-angiotensin-system in neurological disease. Clin Exp Pharmacol Physiol.

[48] Bennion DM, Haltigan E, Regenhardt RW, Steckelings UM, Sumners C (2015). Neuroprotective mechanisms of the ACE2-angiotensin-(1–7)-Mas axis in stroke. Curr Hypertens Rep.

[49] Zhou Z, Kang H, Li S, Zhao X (2020). Understanding the neurotropic characteristics of SARS-CoV-2: from neurological manifestations of COVID-19 to potential neurotropic mechanisms. J Neurol.

[50] Ramani A, Müller L, Ostermann PN (2020). SARS-CoV-2 targets neurons of 3D human brain organoids. EMBO J..

[51] Pellegrini L, Albecka A, Mallery DL (2020). SARS-CoV-2 infects the brain choroid plexus and disrupts the blood-CSF barrier in human brain organoids. Cell Stem Cell.

[52] Iadecola C, Anrather J, Kamel H (2020). Effects of COVID-19 on the nervous system. Cell.

[53] Moore JB, Hune CH (2020). Cytokine release syndrome in severe COVID-19. Science.

[54] Wadman M, Couzin-Frankel J, Kaiser J, Matacic C (2020). A rampage through the body. Science.

[55] Callaway E (2020). The race for coronavirus vaccines: a graphical guide. Nature.

[56] Gao Q, Bao L (2020). Rapid development of an inactivated vaccine candidate for SARS-CoV-2. Science.

[57] Yu J, Tostanoski LH (2020). DNA vaccine protection against SARS-CoV-2 in rhesus macaques. Science.

[58] Wang H, Zhang Y, Huang B (2020). Development of an inactivated vaccine candidate, BBIBP-CorV, with potent protection against SARS-CoV-2. Cell.

[59] Yang J, Wang W, Chen Z (2020). A vaccine targeting the RBD of the S protein of SARS-CoV-2 induces protective immunity. Nature.

[60] Corbett KS, Edwards DK, Leist SR (2020). SARS-CoV-2 mRNA vaccine design enabled by prototype pathogen preparedness. Nature.

[61] Mercado NB, Zahn R, Wegmann F (2020). Single-shot Ad26 vaccine protects against SARS-CoV-2 in rhesus macaques. Nature.

[62] van Doremalen N, Lambe T, Spencer A (2020). ChAdOx1 nCoV-19 vaccine prevents SARS-CoV-2 pneumonia in rhesus macaques. Nature.

[63] Mulligan MJ, Lyke KE, Kitchin N (2020). Phase 1/2 study of COVID-19 RNA vaccine BNT162b1 in adults. Nature.

[64] Sahin U, Muik A, Derhovanessian E (2020). COVID-19 vaccine BNT162b1 elicits human antibody and T H 1 T-cell responses. Nature.

[65] Keech C, Albert G, Cho I (2020). Phase 1–2 trial of a SARS-CoV-2 recombinant spike protein nanoparticle vaccine. N Engl J Med.

[66] Callaway E (2020). COVID vaccine excitement builds as Moderna reports third positive result. Nature.

[67] Callaway E (2020). What Pfizer's landmark COVID vaccine results mean for the pandemic. Nature.

[68] Brouwer PJM, Caniels TG, van der Straten K (2020). Potent neutralizing antibodies from COVID-19 patients define multiple targets of vulnerability. Science.

[69] Ju B, Zhang Q (2020). Human neutralizing antibodies elicited by SARS-CoV-2 infection. Nature.

[70] Pinto D, Park YJ (2020). Cross-neutralization of SARS-CoV-2 by a human monoclonal SARS-CoV antibody. Nature.

[71] Rogers TF, Zhao F, Huang D (2020). Isolation of potent SARS-CoV-2 neutralizing antibodies and protection from disease in a small animal model. Science.

[72] Shi R, Shan C (2020). A human neutralizing antibody targets the receptor binding site of SARS-CoV-2. Nature.

[73] Hansen J, Baum A, Pascal KE (2020). Studies in humanized mice and convalescent humans yield a SARS-CoV-2 antibody cocktail. Science.

[74] Cao Y, Su B, Guo X (2020). Potent neutralizing antibodies against SARS-CoV-2 identified by high-throughput single-cell sequencing of convalescent patients' B cells. Cell.

[75] Baum A, Fulton BO, Wloga E (2020). Antibody cocktail to SARS-CoV-2 spike protein prevents rapid mutational escape seen with individual antibodies. Science.

[76] Baum A, Ajithdoss D, Copin R (2020). REGN-COV2 antibodies prevent and treat SARS-CoV-2 infection in rhesus macaques and hamsters. Science.

[77] Tortorici MA, Beltramello M, Lempp FA (2020). Ultrapotent human antibodies protect against SARS-CoV-2 challenge via multiple mechanisms. Science.

[78] Wu Y, Wang F, Shen C (2020). A noncompeting pair of human neutralizing antibodies block COVID-19 virus binding to its receptor ACE2. Science.

[79] Zost SJ, Gilchuk P, Case JB (2020). Potently neutralizing and protective human antibodies against SARS-CoV-2. Nature.

[80] Hassan AO, James Brett Case JB, Winkler ES (2020). A SARS-CoV-2 infection model in mice demonstrates protection by neutralizing antibodies. Cell.

[81] Chen P, Nirula A, Heller B (2020). SARS-CoV-2 neutralizing antibody LY-CoV555 in outpatients with COVID-19. N Engl J Med..

[82] Jianhui Nie J, Li Q, Wu J (2020). Establishment and validation of a pseudovirus neutralization assay for SARS-CoV-2. Emerg Microbes Infect.

[83] Tan CW, Chia WN, Qin X (2020). A SARS-CoV-2 surrogate virus neutralization test based on antibody-mediated blockage of ACE2-spike protein-protein interaction. Nat Biotechnol..

[84] Monteil V, Kwon H, Prado P (2020). Inhibition of SARS-CoV-2 infections in engineered human tissues using clinical-grade soluble human ACE2. Cell.

[85] Adedeji AO, Severson W, Jonsson C, Singh K, Weiss SR, Sarafianos SG (2013). Novel inhibitors of severe acute respiratory syndrome coronavirus entry that act by three distinct mechanisms. J Virol.

[86] Hofmann-Winkler H, Moerer O, Alt-Epping S (2020). Camostat mesylate may reduce severity of coronavirus disease 2019 sepsis: a first observation. Crit Care Explor..

[87] Breining P, Frølund AL, Højen JF (2020). Camostat mesylate against SARS-CoV-2 and COVID-19-Rationale, dosing and safety. Basic Clin Pharmacol Toxicol..

[88] Glowacka I, Bertram S, Müller MA (2011). Evidence that TMPRSS2 activates the severe acute respiratory syndrome coronavirus spike protein for membrane fusion and reduces viral control by the humoral immune response. J Virol.

[89] Matsuyama S, Nao N, Shirato K (2020). Enhanced isolation of SARS-CoV-2 by TMPRSS2-expressing cells. Proc Natl Acad Sci USA.

[90] Elkin SR, Oswald NW, Reed DK, Mettlen M, MacMillan JB, Schmid SL (2016). Ikarugamycin: a natural product inhibitor of clathrin-mediated endocytosis. Traffic.

[91] McCluskey A, Daniel JA, Hadzic G (2013). Building a better dynasore: the dyngo compounds potently inhibit dynamin and endocytosis. Traffic.

[92] Weir DL, Laing ED, Smith IL, Wang LF, Broder CC (2014). Host cell virus entry mediated by Australian bat lyssavirus G envelope glycoprotein occurs through a clathrin-mediated endocytic pathway that requires actin and Rab5. Virol J.

[93] Wang S, Li W, Hui H (2020). Cholesterol 25-Hydroxylase inhibits SARS-CoV-2 and other coronaviruses by depleting membrane cholesterol. EMBO J..

[94] Dai W, Zhang B, Su H (2020). Structure-based design of antiviral drug candidates targeting the SARS-CoV-2 main protease. Science.

[95] Zhang L, Lin D, Sun X, Curth U, Drosten C, Sauerhering L, Becker S, Rox K, Hilgenfeld R (2020). Crystal structure of SARS-CoV-2 main protease provides a basis for design of improved α-ketoamide inhibitors. Science.

[96] Hillen HS, Kokic G, Farnung L, Dienemann C, Tegunov D, Cramer P (2020). Structure of replicating SARS-CoV-2 polymerase. Nature.

[97] Elfiky AA (2020). Ribavirin, Remdesivir, Sofosbuvir, Galidesivir, and Tenofovir against SARS-CoV-2 RNA dependent RNA polymerase (RdRp): a molecular docking study. Life Sci.

[98] Beigel JH, Tomashek KM (2020). Remdesivir for the treatment of COVID-19—preliminary report. N Engl J Med.

[99] Grein J, Ohmagari N (2020). Compassionate use of remdesivir for patients with severe COVID-19. N Engl J Med.

[100] Cohen J, Kupferschmidt K (2020). 'A very, very bad look' for remdesivir. Science.

[101] Kaur R, Kaur G, Gill RK, Soni R, Bariwal J (2014). Recent developments in tubulin polymerization inhibitors: an overview. Eur J Med Chem.

[102] Devaux CA, Rolain JM, Colson P, Raoult D (2020). New insights on the antiviral effects of chloroquine against coronavirus: what to expect for COVID-19?. Int J Antimicrob Agents.

[103] Blignaut M, Espach Y, van Vuuren M, Dhanabalan K, Huisamen B (2019). Revisiting the cardiotoxic effect of chloroquine. Cardiovasc Drugs Ther.

[104] Torjesen I (2020). Covid-19: Hydroxychloroquine does not benefit hospitalised patients, UK trial finds. BMJ.

[105] Tang W, Cao Z, Han M (2020). Hydroxychloroquine in patients with mainly mild to moderate coronavirus disease 2019: open label, randomised controlled trial. BMJ.

[106] Frlan R, Gobec S (2006). Inhibitors of cathepsin B. Curr Med Chem.

[107] Wyczałkowska-Tomasik A, Pączek L (2012). Cathepsin B and L activity in the serum during the human aging process: cathepsin B and L in aging. Arch Gerontol Geriatr.

[108] Mareti A, Kritsioti C, Georgiopoulos G, Vlachogiannis NI (2019). Cathepsin B expression is associated with arterial stiffening and atherosclerotic vascular disease. Eur J Prev Cardiol.

[109] Keller CW, Loi M, Ligeon LA, Gannagé M, Lünemann JD, Münz C (2018). Endocytosis regulation by autophagy proteins in MHC restricted antigen presentation. Curr Opin Immunol.

[110] Andersen KG, Rambaut A, Lipkin WI, Holmes EC, Garry RF (2020). The proximal origin of SARS-CoV-2. Nat Med.

[111] Zanker D, Chen W (2014). Standard and immunoproteasomes show similar peptide degradation specificities. Eur J Immunol.

[112] Tsakiri EN, Trougakos IP (2015). The amazing ubiquitin-proteasome system: structural components and implication in aging. Int Rev Cell Mol Biol.

[113] Stratford FL, Chondrogianni N, Trougakos IP, Gonos ES, Rivett AJ (2006). Proteasome response to interferon-gamma is altered in senescent human fibroblasts. FEBS Lett.

[114] Bojkova D, Klann K, Koch B, Widera M, Krause D, Ciesek S, Cinatl J, Münch C (2020). Proteomics of SARS-CoV-2-infected host cells reveals therapy targets. Nature.

[115] Zhang K, Meng X, Li D, Yang J, Kong J, Hao P, Guo T, Zhang M, Zhang Y, Zhang C (2015). Angiotensin(1–7) attenuates the progression of streptozotocin-induced diabetic renal injury better than angiotensin receptor blockade. Kidney Int.

[116] Passos-Silva DG, Verano-Braga T, Santos RA (2013). Angiotensin-(1–7): beyond the cardio-renal actions. Clin Sci (Lond).

[117] Patel VB, Zhong JC, Grant MB, Oudit GY (2016). Role of the ACE2/Angiotensin 1–7 axis of the renin-angiotensin system in heart failure. Circ Res.

[118] Bennion DM, Haltigan E, Regenhardt RW, Steckelings UM, Sumners C (2015). Neuroprotective mechanisms of the ACE2-angiotensin-(1–7)-Mas axis in stroke. Curr Hypertens Rep.

[119] Alenina N, Xu P, Rentzsch B, Patkin EL, Bader M (2008). Genetically altered animal models for Mas and angiotensin-(1–7). Exp Physiol.

[120] Varga Z, Flammer AJ, Steiger P, Haberecker M, Andermatt R, Zinkernagel AS, Mehra MR, Schuepbach RA, Ruschitzka F, Moch H (2020). Endothelial cell infection and endotheliitis in COVID-19. Lancet.

[121] Al-Samkari H, Karp Leaf RS, Dzik WH, Carlson JC, Fogerty AE, Waheed A, Goodarzi K, Bendapudi P, Bornikova L, Gupta S, Leaf D, Kuter DJ, Rosovsky RP (2020). COVID and coagulation: bleeding and thrombotic manifestations of SARS-CoV2 infection. Blood.

[122] Terpos E, Ntanasis-Stathopoulos I, Elalamy I, Kastritis E, Sergentanis TN, Politou M, Psaltopoulou T, Gerotziafas G, Dimopoulos MA (2020). Hematological findings and complications of COVID-19. Am J Hematol.

[123] Ramlall V (2020). Immune complement and coagulation dysfunction in adverse outcomes of SARS-CoV-2 infection. Nat Med.

[124] Mathew D (2020). Deep immune profiling of COVID-19 patients reveals distinct immunotypes with therapeutic implications. Science.

[125] Lucas C (2020). Longitudinal analyses reveal immunological misfiring in severe COVID-19. Nature.

[126] Laing AG (2020). A dynamic COVID-19 immune signature includes associations with poor prognosis. Nat Med.

[127] Bonam SR, Srini V, Kaveri SV, Sakuntabhai A, Gilardin L, Bayry J (2020). Adjunct immunotherapies for the management of severely ill COVID-19 patients. Cell Rep Med.

[128] Giamarellos-Bourboulis EJ, Netea MG, Rovina N (2020). Complex immune dysregulation in COVID-19 patients with severe respiratory failure. Cell Host Microbe..

[129] Blanco-Melo D, Nilsson-Payant BE (2020). Imbalanced host response to SARS-CoV-2 drives development of COVID-19. Cell.

[130] Wilk AJ, Rustagi A, Zhao NQ (2020). A single-cell atlas of the peripheral immune response in patients with severe COVID-19. Nat Med.

[131] Bastard P, Rosen LB, Zhang Q (2020). Auto-antibodies against type I IFNs in patients with life-threatening COVID-19. Science.

[132] Zhang Q, Bastard P, Liu Z (2020). Inborn errors of type I IFN immunity in patients with life-threatening COVID-19. Science.

[133] Rydyznski Moderbacher C, Ramirez CI, Dan JM (2020). Antigen-Specific adaptive immunity to SARS-CoV-2 in acute COVID-19 and associations with age and disease severity. Cell.

[134] Zhang X, Tan Y (2020). Viral host factors related to the clinical outcome of COVID-19. Nature.

[135] The RECOVERY Collaborative Group (2020). N Engl J Med.

[136] Le Bert N (2020). SARS-CoV-2-specific T cell immunity in cases of COVID-19 and SARS, and uninfected controls. Nature.

[137] Braun J (2020). SARS-CoV-2-reactive T cells in healthy donors and patients with COVID-19. Nature.

[138] Kupferschmidt K (2020). The pandemic virus is slowly mutating. But does it matter?. Science.

[139] Plante JA, Liu Y, Liu J (2020). Spike mutation D614G alters SARS-CoV-2 fitness. Nature.

[140] Hou YJ, Chiba S, Halfmann P (2020). D614G variant exhibits efficient replication ex vivo and transmission in vivo. Science.

[141] Korber B (2020). Tracking changes in SARS-CoV-2 spike: evidence that D614G increases infectivity of the COVID-19 virus. Cell.

[142] Li Q, Wu J, Nie J (2020). The impact of mutations in SARS-CoV-2 spike on viral infectivity and antigenicity. Cell.

[143] Brest P, Refae S, Mograbi B, Hofman B, Milano G (2020). Host polymorphisms may impact SARS-CoV-2 infectivity. Trends Genet.

[144] Takahashi T (2020). Sex differences in immune responses that underlie COVID-19 disease outcomes. Nature.

[145] Mallapaty S (2020). The coronavirus is most deadly if you are older and male—new data reveal the risks. Nature.

[146] Akbar AN, Gilroy DW (2020). Aging immunity may exacerbate COVID-19. Science.

[147] Liu STH (2020). Convalescent plasma treatment of severe COVID-19: a propensity score-matched control study. Nat Med.

[148] Terpos E, Mentis A, Dimopoulos MA (2020). Loss of anti-SARS-CoV-2 antibodies in Mild Covid-19. N Engl J Med.

[149] Dai L (2020). A universal design of betacoronavirus vaccines against COVID-19, MERS, and SARS. Cell.

[150] Wu S (2020). A single dose of an adenovirus-vectored vaccine provides protection against SARS-CoV-2 challenge. Nat Commun.

[151] Zhang NN (2020). A thermostable mRNA vaccine against COVID-19. Cell.

[152] Edwards KM (2020). Vaccines targeting SARS-CoV-2 tested in humans. Nat Med.

[153] Chi X (2020). A neutralizing human antibody binds to the N-terminal domain of the Spike protein of SARS-CoV-2. Science.

[154] Anna Z, Wec AZ (2020). Broad neutralization of SARS-related viruses by human monoclonal antibodies. Science.

[155] Zost SJ (2020). Rapid isolation and profiling of a diverse panel of human monoclonal antibodies targeting the SARS-CoV-2 spike protein. Nat Med.

[156] Arvin AM (2020). A perspective on potential antibody-dependent enhancement of SARS-CoV-2. Nature.

[157] Chan KK (2020). Engineering human ACE2 to optimize binding to the spike protein of SARS coronavirus 2. Science.

[158] Maisonnasse P (2020). Hydroxychloroquine use against SARS-CoV-2 infection in non-human primates. Nature.

[159] Hoffmann M (2020). Chloroquine does not inhibit infection of human lung cells with SARS-CoV-2. Nature.

[160] Angus DC (2020). Effect of hydrocortisone on mortality and organ support in patients with severe COVID-19: the REMAP-CAP COVID-19 corticosteroid domain randomized clinical trial. JAMA.

[161] Tomazini BM (2020). Effect of dexamethasone on days alive and ventilator-free in patients with moderate or severe acute respiratory distress syndrome and COVID-19: the CoDEX randomized clinical trial. JAMA.

[162] National Library of Medicine (U.S.). (2020 August 30–2022 January 30) Phase III Trial of a COVID-19 vaccine of adenovirus vector in adults 18 years old and above. Identifier: NCT04526990. https://clinicaltrials.gov/ct2/show/NCT04526990.

[163] National Library of Medicine (U.S.). (2020 August 31–2021 May 1) Clinical trial of efficacy, safety, and immunogenicity of Gam-COVID-vac vaccine against COVID-19 (RESIST). Identifier: NCT04530396. https://clinicaltrials.gov/ct2/show/NCT04530396.

[164] National Library of Medicine (U.S.). (2020 July 16–2021 September 16) A study to evaluate the efficacy, safety and immunogenicity of inactivated SARS-CoV-2 vaccines (Vero Cell) in healthy population aged 18 years old and above (COVID-19). Identifier: NCT04510207. https://clinicaltrials.gov/ct2/show/NCT04510207.

[165] National Library of Medicine (U.S.). (2020 July 21–2021 October) Clinical trial of efficacy and safety of Sinovac's adsorbed COVID-19 (inactivated) vaccine in healthcare professionals (PROFISCOV). Identifier: NCT04456595. https://clinicaltrials.gov/ct2/show/NCT04456595.

[166] National Library of Medicine (U.S.). (2020 July 27–2022 October 27) A study to evaluate efficacy, safety, and immunogenicity of mRNA-1273 vaccine in adults aged 18 years and older to prevent COVID-19. Identifier: NCT04470427. https://clinicaltrials.gov/ct2/show/NCT04470427.

[167] Khan A, Benthin C, Zeno B (2017). A pilot clinical trial of recombinant human angiotensin-converting enzyme 2 in acute respiratory distress syndrome. Crit Care.

[168] National Library of Medicine (U.S.). (2020 April 30–2020 November) Recombinant human angiotensin-converting enzyme 2 (rhACE2) as a treatment for patients with COVID-19 (APN01-COVID-19). Identifier: NCT04335136. https://clinicaltrials.gov/ct2/show/NCT04335136.

[169] Liu T, Luo S, Libby P, Shi GP (2020). Cathepsin L-selective inhibitors: a potentially promising treatment for COVID-19 patients. Pharmacol Ther.

[170] National Library of Medicine (U.S.). (2020 September 30–2021 June 15) Angiotensin-(1,7) treatment in COVID-19: the ATCO trial (ATCO). Identifier: NCT04332666. https://clinicaltrials.gov/ct2/show/NCT04332666.

[171] National Library of Medicine (U.S.). (2020 August–2021 December) TXA127 for the treatment of severe COVID-19. Identifier: NCT04401423. https://clinicaltrials.gov/ct2/show/NCT04401423.

[172] Ghazizadeh Z, Majd H, Richter M (2020). Androgen regulates SARS-CoV-2 receptor levels and is associated with severe COVID-19 symptoms in men. bioRxiv.

[173] Hirshburg JM, Kelsey PA, Therrien CA, Gavino AC, Reichenberg JS (2016). Adverse effects and safety of 5-alpha reductase inhibitors (Finasteride, Dutasteride): a systematic review. J Clin Aesthet Dermatol.

[174] Khan N, Patel D, Xie D, Lewis J, Trivedi C, Yang YX (2020). Impact of anti-TNF and Thiopurines medications on the development of COVID-19 in patients with inflammatory bowel disease: a nationwide VA cohort study. Gastroenterology.

[175] Tursi A, Vetrone LM, Papa A (2020). Anti-TNF-α agents in inflammatory bowel disease and course of COVID-19. Inflamm Bowel Dis.

[176] Gerriets V, Bansal P, Goyal A, Khaddour K. Tumor necrosis factor (TNF) inhibitors. In: StatPearls. Treasure Island (FL): StatPearls Publishing; July 4, 2020. 29494032

[177] Chen Y, Lear T, Evankovich J, et al. A high throughput screen for TMPRSS2 expression identifies FDA-approved and clinically advanced compounds that can limit SARS-CoV-2 entry. Res Sq. 2020;rs.3.rs-48659. 10.1038/s41467-021-24156-y PMC8222394 34162861

[178] Scott LJ, Goa KL (2000). Verteporfin. Drugs Aging.

[179] Lindauer M, Hochhaus A (2018). Dasatinib. Recent results. Cancer Res.

[180] Venetoclax. In: LiverTox: Clinical and research information on drug-induced liver injury. Bethesda (MD): National Institute of Diabetes and Digestive and Kidney Diseases; April 4, 2017. 31643176

[181] Hoffmann M, Schroeder S, Kleine-Weber H, Müller MA, Drosten C, Pöhlmann S (2020). Nafamostat mesylate blocks activation of SARS-CoV-2: new treatment option for COVID-19. Antimicrob Agents Chemother.

[182] Chen X, Xu Z, Zeng S (2019). The molecular aspect of antitumor effects of protease inhibitor nafamostat mesylate and its role in potential clinical applications. Front Oncol.

[183] Deng L, Li C, Zeng Q (2020). Arbidol combined with LPV/r versus LPV/r alone against corona virus disease 2019: a retrospective cohort study. J Infect.

[184] Javorac D, Grahovac L, Manić L (2020). An overview of safety assessment of the medicines currently used in the treatment of COVID-19 disease. Food Chem Toxicol.

[185] Plaze M, Attali D, Petit AC (2020). Repurposing chlorpromazine to treat COVID-19: the reCoVery study. Encephale.

[186] Adams CE, Awad GA, Rathbone J, Thornley B, Soares-Weiser K. Chlorpromazine versus placebo for schizophrenia. Cochrane Database Syst Rev. 2014;CD000284. 10.1002/14651858.CD000284.pub3 PMC10640712 24395698

[187] Shamsi A, Mohammad T, Anwar S (2020). Glecaprevir and Maraviroc are high-affinity inhibitors of SARS-CoV-2 main protease: possible implication in COVID-19 therapy. Biosci Rep..

[188] Shiffman ML (2004). Side effects of medical therapy for chronic hepatitis C. Ann Hepatol.

[189] Okonkwo RI, Weidmann AE, Effa EE (2016). Renal and bone adverse effects of a tenofovir-based regimen in the treatment of HIV-infected children: a systematic review [published correction appears in Drug Saf. 2016 Apr; 39(4):369]. Drug Saf.

[190] Yousefi B, Valizadeh S, Ghaffari H, Vahedi A, Karbalaei M, Eslami M (2020). A global treatments for coronaviruses including COVID-19. J Cell Physiol..

[191] Deftereos SG, Giannopoulos G, Vrachatis DA (2020). Effect of colchicine vs standard care on cardiac and inflammatory biomarkers and clinical outcomes in patients hospitalized with coronavirus disease 2019: the GRECCO-19 randomized clinical trial. JAMA Netw Open.

[192] Gautret P, Lagier JC, Parola P (2020). Hydroxychloroquine and azithromycin as a treatment of COVID-19: results of an open-label non-randomized clinical trial. Int J Antimicrob Agents.

[193] Baron SA, Devaux C, Colson P, Raoult D, Rolain JM (2020). Teicoplanin: an alternative drug for the treatment of COVID-19?. Int J Antimicrob Agents.

[194] Mendonca P, Soliman KFA (2020). Flavonoids activation of the transcription factor Nrf2 as a hypothesis approach for the prevention and modulation of SARS-CoV-2 infection severity. Antioxidants (Basel).

[195] Wu J (2020). Tackle the free radicals damage in COVID-19. Nitric Oxide.

[196] Finzi E (2020). Treatment of SARS-CoV-2 with high dose oral zinc salts: a report on four patients. Int J Infect Dis.

[197] Cheah IK, Halliwell B (2020). Could ergothioneine aid in the treatment of coronavirus patients?. Antioxidants (Basel).

[198] Guaraldi G, Meschiari M, Cozzi-Lepri A (2020). Tocilizumab in patients with severe COVID-19: a retrospective cohort study. Lancet Rheumatol.

[199] Montesarchio V, Parrela R, Iommelli C (2020). Outcomes and biomarker analyses among patients with COVID-19 treated with interleukin 6 (IL-6) receptor antagonist sarilumab at a single institution in Italy. J Immunother Cancer.

[200] Zhou F, Liu YM, Xie J (2020). Comparative impacts of ACE (angiotensin-converting enzyme) inhibitors versus angiotensin II receptor blockers on the risk of COVID-19 mortality. Hypertension.

[201] Pranata R, Permana H, Huang I (2020). The use of renin angiotensin system inhibitor on mortality in patients with coronavirus disease 2019 (COVID-19): a systematic review and meta-analysis. Diabetes Metab Syndr.

[202] Guo X, Zhu Y, Hong Y (2020). Decreased mortality of COVID-19 with renin-angiotensin-aldosterone system inhibitors therapy in patients with hypertension: a meta-analysis. Hypertension.

[203] Dworakowska D, Grossman AB (2020). Renin-angiotensin system inhibitors in management of hypertension during the COVID-19 pandemic. J Physiol Pharmacol.

